# Effects of Harmless Municipal Solid Waste Incineration Fly Ash on the Macroscopic Properties and Microstructure of Recycled Aggregate Concrete

**DOI:** 10.3390/ma18081866

**Published:** 2025-04-18

**Authors:** Bochen Song, Yefan Li, Wengang Zhang

**Affiliations:** 1 School of Civil Engineering and Geomatics, Shandong University of Technology, Zibo 255000, China; 22110902093@stumail.sdut.edu.cn; 2 The Fourth Construction Co., Ltd. of China Construction Eighth Engineering Division, Qingdao 266000, China; 15264343897@163.com

**Keywords:** RCA, HMSWIFA, compressive strength, flexural strength, frost resistance, microstructure

## Abstract

With the increasing rate of urbanization and the annual rise in municipal domestic waste, the use of harmless municipal solid waste incineration fly ash (HMSWIFA) as a construction material has been gradually adopted and promoted. However, significant differences exist in how various characteristics of HMSWIFA affect the performance of recycled aggregate concrete (RCA). To analyze the effects of HMSWIFA content and particle size on the macroscopic properties and microstructure of RCA, this paper conducts compressive, flexural, frost resistance, and Scanning Electron Microscope (SEM) characterization on RCA with varying dosages and particle sizes of HMSWIFA as a cement replacement. The results indicate that HMSWIFA enhances the compressive strength (CS) and frost resistance of RCA. Experimental data reveal that HMSWIFA with a particle size of 600–900 μm exhibits the best modification effect at an admixture level of 10–15%: the 28-day CS increased by 1.90–3.60%, the mass loss after freezing and thawing decreased by 0.37–0.45%, and the increase in dynamic elastic modulus reached 16.09–16.44%. Notably, the flexural strength (FS) experienced a reduction of 1.81% at a high dosage of the optimal particle size. This study elucidates the coupling relationship of “particle size-admixture-performance” in HMSWIFA-modified recycled concrete, demonstrating that reasonable control of the particle size distribution of HMSWIFA can achieve a synergistic effect of mechanical enhancement and durability improvement. The research findings provide a valuable reference for the application of municipal waste incineration HMSWIFA in RCA, facilitating the recycling of waste resources to mitigate pollution and enhance energy efficiency.

## 1. Introduction

Amidst China’s meteoric socioeconomic advancement, the national economy is expanding, leading to an increase in consumption levels and, consequently, a growing amount of urban waste. According to statistics, in 2018, China’s urban waste removal reached 2.28 million tons, an increase of 7100 tons compared to 2009 [1]. High-volume fly ash is generated during the incineration of urban domestic waste, which poses ecological risks if it contaminates groundwater, the surrounding environment, or the atmosphere. At present, the treatment of municipal waste is broadly divided into two kinds: one is to treat it (mainly incineration) to landfill, and the other is to realize the reuse of resources. Domestic waste through incineration landfills this way to a certain extent to solve the problem of harmless treatment of domestic waste in China, but also in a sustainable way to provide power energy. However, China’s incineration of waste treatment is still to be perfected and improved, especially in the domestic waste incineration of dioxins and other toxic and harmful substances included in the “National Hazardous Waste List”. However, with the continuous development of urbanization and increasing population exacerbating the daily production of municipal waste incineration fly ash (MSWIFA), at the same time the reduction of landfill sites and the increasingly strict environmental policy make incineration landfill treatment not a long-term viable method [2], so there is an urgent need for a new and environmentally friendly method of handling MSW incineration fly ash.

Resource reuse is a prominent research focus in the field of construction engineering, holding significant implications for energy conservation and environmental protection. Given the physicochemical properties and economic value of fly ash, harmlessly treated fly ash can be utilized in concrete as a raw material for cement production or as a cement substitute [3,4]. Alderete attempted to replace cement by grinding, heating, and washing fly ash, successfully designing concrete with favorable properties. However, this treatment method is more complex, has a greater environmental impact, and incurs higher costs, making large-scale applications in engineering challenging [5]. Saikia explored the direct use of fly ash as a fine aggregate; however, fly ash contains trace elements such as metallic aluminum, along with significant amounts of sulfates and soluble salts. These constituents can release hydrogen gas (H_2_) and simultaneously increase the production of expansion products like ettringite, which significantly undermines the structural integrity of mortar matrices. As a result, the direct use of fly ash as a fine aggregate in concrete applications shows limited technical feasibility [6]. Previous studies have shown that cold bonding pelletization techniques provide an effective method for recycling fine particulate fractions derived from municipal solid waste incineration bottom ash, offering a viable pathway for sustainable material recovery [7]. Tang [8] utilized cold-bonded pelletization to convert fly ash, in conjunction with supplementary waste streams and industrial byproducts, into engineered lightweight aggregates (ELAs). These aggregates were subsequently incorporated as sustainable components in concrete formulations. Furthermore, cold-bonded granulation technology for producing artificial lightweight aggregates offers advantages such as low energy consumption, reduced carbon emissions, cost-effectiveness, and minimal environmental impact [9].

In addition, Lu et al. investigated the effects of substituting natural river sand with fly ash at varying rates during freeze-thaw (F-T) cycles on the macroscopic performance, micromorphology, and pore structure of concrete. The results indicated that the incorporation of fly ash effectively refined the pore structure of the concrete specimens. However, the addition of fly ash resulted in increased porosity and decreased density during the F-T cycles, ultimately leading to a reduction in concrete strength. Higher fly ash content exacerbated these detrimental effects and diminished the durability of the concrete, particularly its frost resistance [10]. Liu and Tang found that fly ash adversely affects cement hydration, reducing both the total heat release and the strength of the mortar. Excessive fly ash content can lead to insufficient cement-water reactions due to the high water absorption rate of fly ash and the presence of aluminum metal. In an alkaline environment, the alumina coating on the surface of the aluminum dissolves, resulting in hydrogen generation from direct contact between the aluminum and the solution, which can cause cracks in the concrete [11]. Liu recycled fly ash by manufacturing artificial light coarse aggregates (ALCAs) and partially replacing natural aggregates (NAs) with these ALCAs to create green concrete. It was found that when fly ash was added to the ALCAs, the leaching values of copper (Cu), lead (Pb), and chromium (Cr) were significantly reduced. The leaching process of all heavy metals complied with the requirements set by the US EPA, leading to the conclusion that ALCAs effectively immobilize heavy metals in fly ash [12]. Chen et al. reported significant differences in the elemental composition of fly ash based on particle size, noting that smaller fly ash particles contained a higher proportion of calcium and a lower proportion of silica [13]. Furthermore, there were notable differences in the heavy metal leaching content among fly ash particles of varying grain sizes, with finer particles exhibiting higher levels of heavy metal leaching [14,15]. Some researchers have found that a smaller percentage of fly ash contains more aluminum, which can negatively impact the mechanical properties of the material [16]. Ala Abu Taqa and his research collaborators [17] systematically investigated the impact of basalt fiber reinforcement on three essential aspects of eco-efficient self-consolidating concrete (SCC): (i) fresh-state rheological properties, (ii) hardened-state load-bearing capacity, and (iii) microscale morphological changes. The binder system effectively examined combined coal combustion byproducts (fly ash) with processed asphalt fines as partial substitutes for traditional cementitious materials. Concrete specimen blocks were prepared using fly ash (FA) with nanosilica (NS) as a partial replacement for the binder, as conducted by Grzegorz Ludwik Golewski [18]. The results indicated that the mechanical properties of the concrete improved at specific substitution rates. Ordinary Portland Cement (OPC) is the most commonly used solidification/stabilization (S/S) binder and is employed for the safe disposal of municipal solid waste fly ash [19]. Currently, both domestic and international research on fly ash-containing RCA, particularly utilizing construction waste, is still in its early stages, highlighting the urgent need for further investigation in this area.

This paper presents a comprehensive study on the design of three different particle sizes of HMSWIFA (100–300 μm, 300–600 μm, and 600–900 μm) and four varying mixing proportions (0%, 10%, 15%, and 20%) incorporated into RCA. A control group of RCA without HMSWIFA was also included for comparison. The study involved conducting compression, flexural, and F-T tests, along with microstructural analysis of the damaged surfaces of the RCA using scanning electron microscopy. Revealing the effect of HMSWIF on the macromechanics and microstructure of RCA, the research findings establish a scientific foundation for advancing the utilization of HMSWIFA in RCA applications, thereby supporting sustainable material innovation in urban construction practices.

## 2. Materials and Methods

### 2.1. Raw Materials

**Figure 1:**
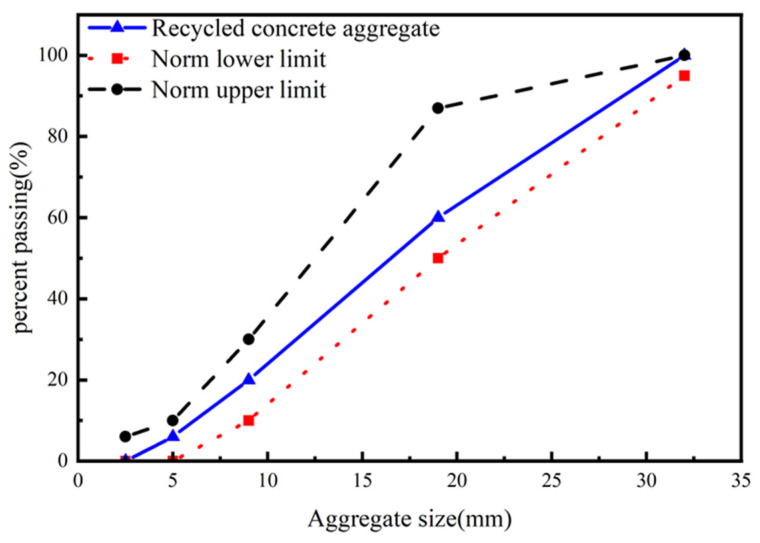
The gradation curve of the coarse aggregates.

Ordinary silicate cement PO42.5 (Zhangdian Shaoying Building Materials Business Department Zibo, China) and recycled concrete aggregate serve as the raw materials for the reference group. In contrast, the raw materials for the RCA made with HMSWIFA include recycled concrete aggregate, natural river sand (Zhangdian Shaoying Building Materials Business Department Zibo, China), ordinary silicate cement PO42.5, and municipal solid waste incineration fly ash (non-hazardous treatment, heavy metal-free). The PO42.5 ordinary silicate cement complies with the specifications outlined in Silicate Cement GB175-2007 (Zhangdian Shaoying Building Materials Business Department Zibo, China) [20]. The specific physical properties and chemical composition are presented in Table 1 and Table 2, respectively. The particle sizes of domestic waste incineration HMSWIFA are categorized as 100–300 μm, 300–600 μm, and 600–900 μm. The ordinary coarse aggregate consists of natural crushed stone, and its specific properties are detailed in Table 3. The recycled aggregates are derived from discarded concrete blocks, which have been processed through a jaw crusher (HONGXING MACHINERY GROUP, Zhengzhou, China), screened with a vibrating screen (Jiangxi Victor International Mining Equipment Co., Ltd., Ganzhou, China), and further refined using a manual crusher (Jiangxi Victor International Mining Equipment Co., Ltd., Ganzhou, China). The recycled aggregate, composed of waste concrete blocks, is crushed by a jaw crusher, screened with a vibrating sieve machine (Jiangxi Victor International Mining Equipment Co., Ltd., Ganzhou, China), and then manually crushed and graded into a continuous range of 5–26.5 mm for recycled coarse aggregate. The gradation curve is illustrated in Figure 1. The fine aggregate is natural river sand, and its physical properties are presented in Table 4.

### 2.2. Mix Proportion

In this experiment, RCA incorporating HMSWIFA was prepared by mixing HMSWIFA with other components. The study investigated the effects of varying HMSWIFA amounts and particle sizes, as well as curing time, regarding the macro-mechanical characteristics, microstructure, and durability of RCA. The principle of single-variable experimentation was employed in designing the mixing ratios. Specifically, while maintaining a constant water–cement ratio, aggregate–cement ratio, sand content, and replacement rate of recycled aggregates, only the amounts, particle sizes, and curing times of the HMSWIFA were altered in the test specimens.

**Figure 2:**
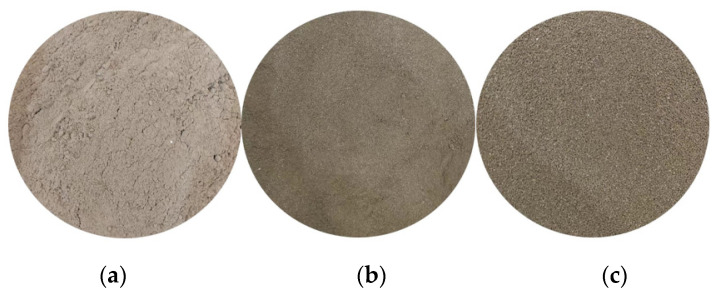
HMSWIFA-RCA of different fineness with the diameter of (**a**) 100~300 μm; (**b**) 300~600 μm; and (**c**) 600~900 μm.

The concrete mixture design protocol was meticulously developed in accordance with the Technical Specifications for Proportioning Design of Conventional Concrete (JGJ 55-2019) [21], C50 ordinary concrete serves as the strength benchmark for proportioning design. The water–cement ratio is set at 0.38. In the test, 20% of recycled aggregate, by equal mass, is used to replace natural crushed stone. Additionally, HMSWIFA is incorporated by replacing an equal mass of cement, with substitution rates of 0%, 10%, 15%, and 20%. These substitution rates are denoted as H0, H10, H15, and H20, respectively. The particle sizes of the HMSWIFA are categorized as 100–300 μm, 300–600 μm, and 600–900 μm, referred to as I, II, and III, respectively, as illustrated in Figure 2a–c below. For instance, I-H10 indicates the RCA made with HMSWIFA of 100–300 μm particle size and a mixing amount of 10%. The specific mixing ratios are shown in Table 5.

### 2.3. Specimen Preparation

Stability and Strength of Concrete with Recycled Aggregate from Construction Waste Due to the high porosity and water absorption of recycled aggregates, poor construction techniques can lead to a reduction in the strength and stability of concrete [22]. To minimize the adverse effects of recycled aggregates, they were first soaked for 24 h, then removed and allowed to dry for approximately 2 h to achieve a saturated surface-dry state [23]. In this test, the dry mixing method was employed. The mixing protocol commenced with the metered addition of Portland cement and HMSWIFA into the blender. This was followed by an initial blending phase lasting 1.5 ± 0.5 min to ensure homogeneous dispersion of the binder components before introducing the liquid phase. Next, natural river sand, natural gravel, and recycled coarse aggregate (Zhangdian Shaoying Building Materials Business Department Zibo, China) were added, followed by dry mixing for 3–5 min to ensure material homogeneity, and subsequent water addition with thorough mixing to achieve complete hydration activation. The mixed concrete was then loaded into the test mold (Dingfeng Surveying and Mapping Instrument Company, Linyi, China), where it was vibrated, and the surface was smoothed to a uniform level. To prevent water evaporation, the surface was covered with plastic film (Dingfeng Surveying and Mapping Instrument Company, Linyi, China). After 24 h, the specimens were demolded. Following specimen labeling, the test units were systematically transferred to a climate-regulated curing facility maintaining precise environmental conditions of 20 ± 2 °C and ≥95% relative humidity (RH) to execute standardized hydration control protocols. In the preparation of SEM samples, after selecting the cement stone specimen, the surface should be cleaned with alcohol (75% ,Bangjin Flagship Store, Zibo, China) and then placed in an oven (Dingfeng Surveying and Mapping Instrument Company, Linyi, China) to dry. Additionally, since the cementite specimen is not electrically conductive, it must be coated with gold before undergoing scanning electron microscope (SEM) (Shangrao Osbarton Optical Instrument Co., Ltd., Shangrao, China) characterization.

### 2.4. Test Methods

**Figure 3:**
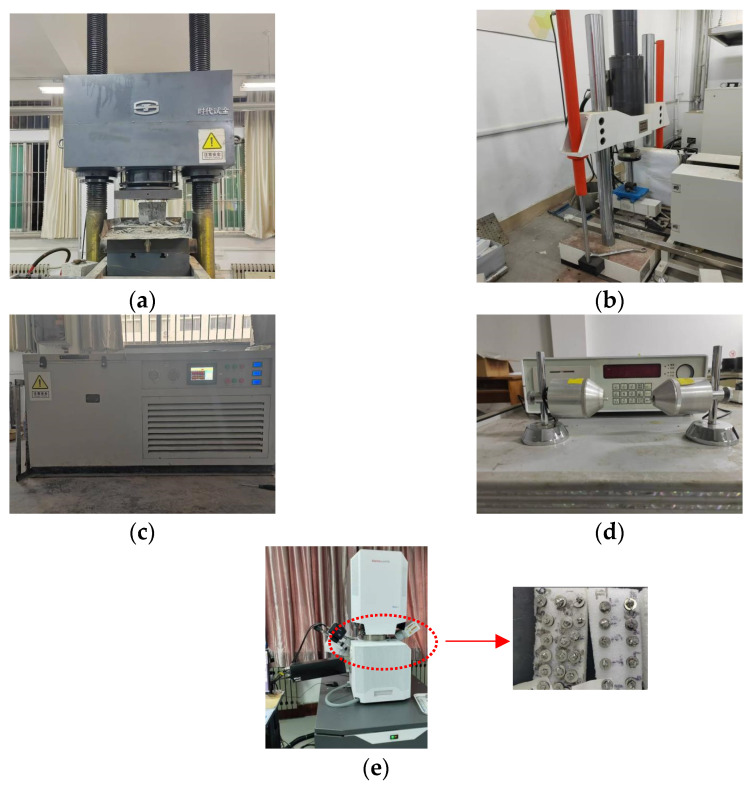
Test instrument: (**a**) CS test; (**b**) FS test; (**c**) F-T cycle test machine; (**d**) dynamic elastic modulus tester; (**e**) Quanta 250 scanning electron microscope.

The fundamental mechanical characterization included an evaluation of compressive resistance and an assessment of flexural capacity, conducted in strict accordance with the Standard for Test Methods of Physical and Mechanical Properties of Concrete (GB/T 50081-2019) [24]. The basic durability tests mainly consist of F-T cycle tests and dynamic elastic modulus determination tests, referencing the Standard for Long-Term Properties and Durability of Ordinary Concrete (GB/T 50082-2009) [25]. Additionally, a scanning electron microscope test using the Quanta 250 (FEI Company, Hillsboro, Oregon) field emission environmental scanning electron microscope allows for the observation of the morphological characteristics of cement stone specimens. The specific sizes of the test specimens and the instruments used for these tests are detailed in Table 6 below, while the specific test instruments are illustrated in Figure 3 below.

## 3. Results and Discussion

### 3.1. Mechanical Properties Analysis

#### 3.1.1. Compressive Strength

**Figure 4:**
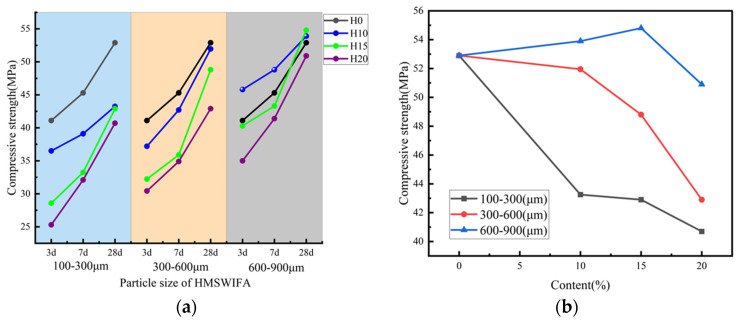
(**a**) Relationship between CS of recycled concrete and different fineness HMSWIFA content; (**b**) influence of HMSWIFA with different content and fineness on CS of recycled concrete.

The variation in the CSof RCA with different HMSWIFA particle sizes (100–300 μm, 300–600 μm, and 600–900 μm) and HMSWIFA admixture levels (0%, 10%, 15%, and 20%) is illustrated in Figure 4a. The results indicate that the CS of RCA increases linearly with extended curing time. However, it decreases linearly with an increase in admixture for HMSWIFA particle sizes of 100–300 μm and 300–600 μm. In contrast, for HMSWIFA particle sizes of 600–900 μm, the CS continues to increase linearly with the increase in curing time.

Figure 4b illustrates the variation in CS of RCA mixtures incorporating HMSWIFA, which contain different content levels and particle gradations, after 28 days of hydration under standard curing conditions. Compared to RCA without HMSWIFA, the CS of RCA containing HMSWIFA with a particle size of 100–300 μm and content of 10%, 15%, and 20% exhibited a gradually decreasing trend, with reductions of 18.2%, 18.9%, and 23.1%, respectively. In contrast, the CS of RCA with HMSWIFA particle sizes of 300–600 μm and content of 10%, 15%, and 20% decreased by 1.0% for each content. When the particle size of the HMSWIFA increased to 600–900 μm, the CS initially increased before declining with higher content. Specifically, at content of 10% and 15%, the CS increased by 1.90% and 3.60%, respectively. However, at a content of 20%, the strength of the RCA began to decline, decreasing by 3.77%. The reason may be closely related to the physical and chemical properties of HMSWIFA itself. HMSWIFA can function as a micro-aggregate in concrete due to its small particle size. When HMSWIFA has an appropriate particle size, it can optimize the gradation of concrete and fill larger pores and cracks in recycled aggregates, thereby refining the pore structure of the concrete and enhancing its mechanical properties. However, HMSWIFA particles contain aluminum, which can lead to the generation of additional hydrogen (H_2_) during the hardening process of concrete. This chemical interaction mechanism may induce coarsening of the pore structure within the cementitious matrix, along with detrimental changes in bulk density characteristics. It is important to note that HMSWIFA particles with smaller sizes typically contain relatively higher amounts of aluminum, which can further exacerbate the hydrogen generation reaction, resulting in increased porosity and decreased density [26]. At the same time, the HMSWIFA has a higher content of CaO, which is a gelling active component, while the content of Al_2_O_3_ and SiO_2_ is less, and the gelling activity is weaker. Therefore, with the increasing admixture of HMSWIFA, the content of silica and aluminum elements decreases, and it is not possible to form more hydrated calcium silicate (alumina) (C(-A)-S-H) gel and hydrated sodium silicate–aluminate (N-A-S-H) gel, which leads to a gradual decrease in the CS of RCA [27].

**Figure 5:**
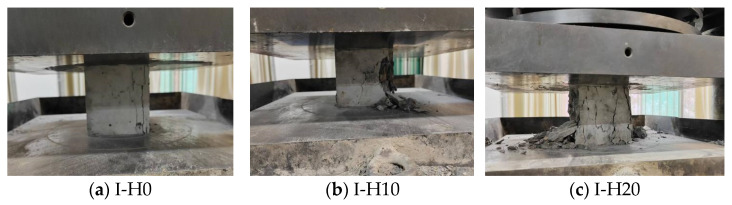
HMSWIFA—RCA cement concrete compressive failure mode. (**a**) I-H0; (**b**) I-H10; (**c**) I-H20.

Systematic variation of HMSWIFA mixed RCA specimens exhibited distinct behaviors during the compressive test. For the benchmark specimen group without HMSWIFA, the initial loading did not result in any noticeable cracks, and the specimens maintained their integrity. Under progressively increasing loading, fine cracks initiated on the specimen surface; however, the macroscopic geometry retained structural integrity without significant deformation. When the load approached the destructive strength, the specimens suddenly failed with a loud noise, causing a significant number of stones to detach from the surface. The failure mode was characterized by a hoop-like formation, indicative of typical brittle damage. For the specimens with lower HMSWIFA content, the damage form and damage process are similar to the specimens of the benchmark group without adding HMSWIFA. With the increasing amount of HMSWIFA, the specimen did not exhibit any obvious cracks during the initial loading stage, and its performance was comparable to that of the benchmark group. However, as the applied load progressively increased, incipient fissure networks began to manifest on the surface of the specimen. These cracks rapidly expanded, accompanied by a distinct rupture sound, as the specimen started to detach from its surface. As the load approached the destructive strength, the specimen emitted a dull sound, indicating significant damage. The damage manifested as severe brittleness, and the destruction process was similar to that of the specimen without the addition of HMSWIFA. The damage pattern is more serious, showing serious brittle damage. Figure 5 shows the damage pattern of specimens with different content of HMSWIFA.

#### 3.1.2. Flexural Strength

**Figure 6:**
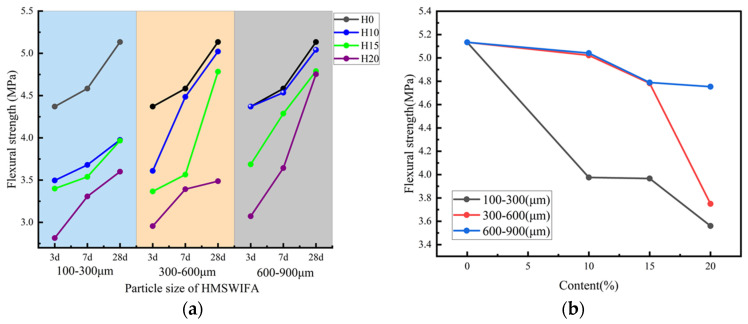
(**a**) Relationship between FS of recycled concrete and different fineness HMSWIFA content. (**b**) Influence of HMSWIFA with different content and fineness on FS of recycled concrete.

The variation in the FS of RCA under different curing times, considering HMSWIFA particle sizes (100–300 μm, 300–600 μm, and 600–900 μm) and HMSWIFA admixture, is illustrated in Figure 6a. The results indicate that the FS of RCA increases with longer curing times for all HMSWIFA particle sizes. However, it decreases linearly as the amount of admixture increases.

Figure 6b illustrates the development of FS in recycled aggregate concrete specimens incorporating HMSWIFA, which contain varying dosage levels and particle gradations. These specimens were subjected to standardized curing conditions over a 28-day hydration period. Compared to RCA mixed with HMSWIFA, the FS of RCA containing HMSWIFA with a particle size of 100–300 μm and mixing amounts of 10%, 15%, and 20% decreased by 22.56%, 22.73%, and 29.88%, respectively. In contrast, the FS of RCA with HMSWIFA particle sizes of 300–600 μm and mixing amounts of 10%, 15%, and 20% decreased by 2.19%, 6.19%, and 6.88%, respectively. For HMSWIFA with a particle size of 600–900 μm, the FS of RCA decreased by 2.19%, 6.84%, and 32.08% for mixing amounts of 10%, 15%, and 20%, respectively. The reason may be that when the amount of HMSWIFA content is small, the overall structure and hydration products of HMSWIFA-RCA concrete specimens are not significantly affected, maintaining a relatively dense three-dimensional mesh cementitious structure, which contributes to high structural stability. However, as the amount of HMSWIFA increases, the number of hydration products gradually decreases, leading to a looser internal structure becoming loose, and a reduction in overall structural stability [28]. The incorporation of HMSWIFA introduces chloride ions into the matrix, where a portion reacts with hydration products to form Friedel’s salt (C_2_A·CaCl_2_·10H_2_O). This phase formation increases both the tortuosity and porosity of internal pores in HMSWIFA-based RCA [29]. Furthermore, if heavy metal ions are present, they interact with hydroxyl ions in the geopolymer reaction system, generating hydroxyl-metal complexes. These complexes reduce the alkalinity of the geopolymer binder and elevate its viscosity, thereby suppressing the dissolution of silico-aluminate precursors. Additionally, the metal–hydroxyl complexes interfere with the polycondensation of SiO_4_ and AlO_4_ tetrahedra, this mechanism additionally impedes the development of aluminosilicate network frameworks and deteriorates the cohesive integrity within the gel matrix [30].

### 3.2. F-T Cycles Test

#### 3.2.1. F-T Test Phenomenon

**Figure 7:**
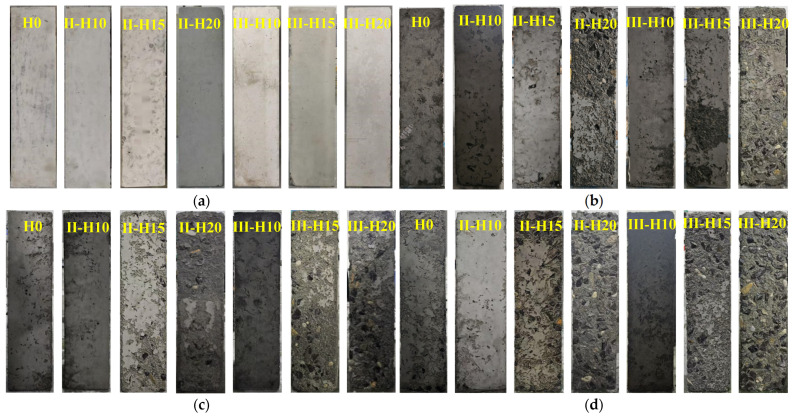

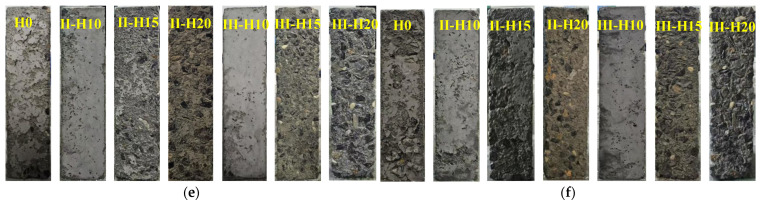
Apparent phenomena of concrete after freeze-thaw cycles: (**a**) 0 freeze-thaw cycles, (**b**) 10 freeze-thaw cycles, (**c**) 30 freeze-thaw cycles, (**d**) 50 freeze-thaw cycles, (**e**) 70 freeze-thaw cycles, and (**f**) 90 freeze-thaw cycles.

The concrete specimens from different groups demonstrated significant variations in frost resistance performance after undergoing various numbers of F-T cycles, as illustrated in Figure 7. Prior to F-T exposure, all samples exhibited uniformly smooth surfaces with no discernible morphological differences. However, after 10 cycles of F-T conditioning, notable distinctions emerged among the groups. When the content of HMSWIFA is 0% and 10%, small holes appear on the surface of the specimen, but there are no significant changes. In the two groups with a 15% HMSWIFA content, the surface of the cement mortar exhibits varying degrees of shedding. The II-H15 group shows slight shedding in some areas, while the III-H15 group experiences a more severe shedding phenomenon. The surface mortar deterioration becomes more pronounced at a HMSWIFA content of 20%. In the II-H20 group, nearly half of the surface area exhibits shedding, whereas the III-H20 group shows even more severe deterioration, with approximately three-quarters of the area affected. Additionally, a small amount of aggregate has become exposed on the surface. After 30 F-T cycles, the benchmark group exhibited a noticeable increase in small holes on its surface, accompanied by some mortar detachment. In contrast, the two groups of specimens with 10% HMSWIFA content maintained a relatively good surface condition, with only a few small holes. However, in the two groups with 15% HMSWIFA, the occurrence of broken rings was exacerbated; the mortar on the surface of the II-15 group began to detach gradually, exposing a small amount of aggregate. The III-H15 group showed a significant increase in surface deterioration, with more aggregate becoming exposed. The detachment phenomenon in the III-H15 group was markedly pronounced, leading to a greater exposure of aggregate on the specimen’s surface. When the HMSWIFA content increased to 20%, the cement mortar on the surface of the II-20 group was nearly completely detached, with only a minimal amount remaining adhered to the specimen. The degree of destruction of the III-H20 group is further increased, and the aggregate exposed on the surface of the specimen is gradually increased. When the F-T cycle is increased to 50 times, the surface of the benchmark group locally shows obvious cement mortar shedding, and the two groups of specimens doped with 10% still have good integrity; the surface damage of the two groups of specimens doped with 15% is further aggravated, the surface cement mortar continues to be shed, and the aggregates are gradually exposed, and the damage of the III-H15 group is still more significant; the surface cement mortar of the two groups of specimens doped with 20% of HMSWIFA is completely shed, and the aggregates are loose, and the marginal aggregates are gradually increased. After aggregate loosening, edge aggregate began to fall off, and F-T loss continued to accumulate. When the F-T cycle 70 times 90 times, the cement mortar of the benchmark group is close to 1/2 of the area slagging off, and the two groups with 10% content begin to appear local slagging off phenomenon; the two groups of specimens with 15% content are further damaged, and it is still more significant for the III-H15 group; the two groups of specimens with 20% content are gradually shedding the surface aggregates, and the edge of the III-H20 group is even more serious in terms of the phenomenon of aggregates shedding.

The reasons for the aforementioned situation are as follows: According to the hydrostatic pressure theory [31] and the osmotic pressure theory [32], when freezing begins, the specimen, being fully saturated with water, allows water to progress through the tiny pores on its surface into its interior. Due to the principles of thermal contraction and the expansion of water at low temperatures, the water within the internal pores expands, which compresses the inner walls of the pores and causes internal damage to the concrete. As melting commences, the ice within the pore spaces melts, and while the internal pore water and broken fine particles may remain trapped inside the specimen, the water in the surface pore spaces carries away the broken fine particles. This results in some specimens exhibiting signs of material loss and detachment. In the case of RCA made with HMSWIFA, a small amount of HMSWIFA admixture can effectively refine the pore structure and micro-defects in the concrete, thereby improving the pore structure and densification of the material. This enhancement reduces the infiltration of environmental water molecules, which in turn decreases the pressure in the interfacial transition zone and improves the F-T resistance of the specimens. However, when an excessive amount of HMSWIFA is added, the opposite effect occurs, leading to increased F-T damage and reduced frost resistance in the concrete specimens.

#### 3.2.2. Mass Loss

**Figure 8:**
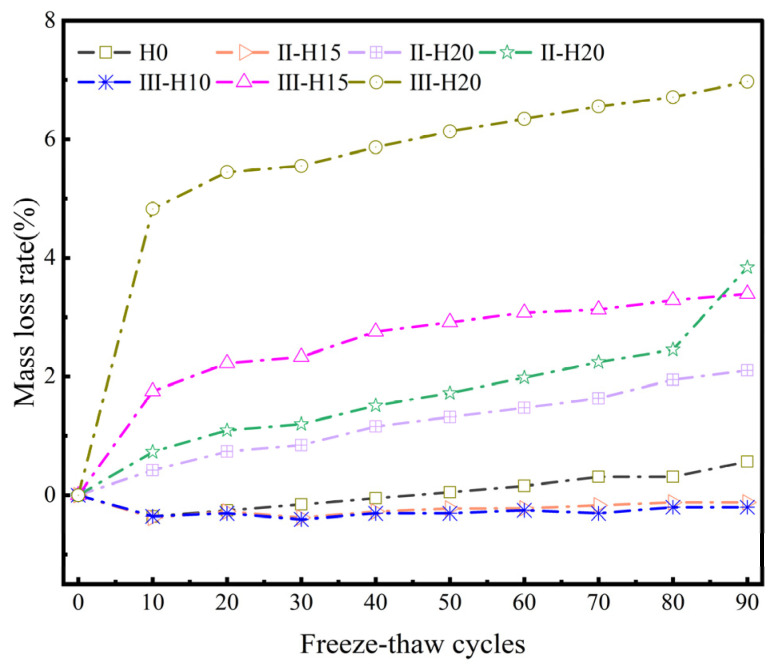
Mass loss rate of concrete specimens under different F-T cycles.

The quality loss of each group of concrete specimens after every 10 freezing and thawing cycles, over a total of 90 cycles, is illustrated in Figure 8. This figure clearly shows that the benchmark group of specimens initially experiences an increase in quality, followed by a subsequent decline. The specimen group containing 10% HMSWIFA (High Modulus Silica Water-Reducing and Internal Curing Admixture) demonstrates a slight increase in quality as the number of freezing and thawing cycles increases, without any significant decline, and remains in a stable state characterized by minor fluctuations. Experimental groups with 15% and 20% fly ash incorporation demonstrated a progressive increase in mass loss that correlated directly with the cumulative increments of F-T cycles. The underlying reason for these observations can be analyzed as follows: during the F-T cycle process, the F-T damage causes the cement mortar on the surface of the concrete specimens to gradually detach, resulting in a reduction in quality [33,34]. With the gradual formation of internal pores and cracks, concrete will absorb external aqueous solutions, which can lead to a decrease in quality [35]. A small amount of HMSWIFA admixture effectively refines the structural pores of the concrete, enhancing the compactness of the specimens, reducing the ingress of water molecules from the external environment, and improving the frost resistance of the specimens [10]. However, when the HMSWIFA admixture exceeds a certain threshold, it can lead to increased porosity in the concrete, thereby reducing its density and frost resistance. An excessive amount of HMSWIFA exacerbates this adverse effect, as the hydration reaction produces a significant quantity of calcium aluminate. The formation of calcium aluminate crystals generates internal pressure within the concrete [36]. If this stress surpasses the tensile strength of the concrete, internal cracks will develop, further diminishing the frost resistance of the concrete specimens. For the baseline group and the 10% HMSWIFA admixture group, the mass of external aqueous solution absorbed during a lesser number of F-T cycles is greater than the mass of cement mortar spalling, resulting in an overall increase in mass. In contrast, the 15% and 20% HMSWIFA admixture groups experience a decrease in internal structural frost resistance due to the excessive HMSWIFA content. This deterioration exacerbates damage during F-T cycles, leading to noticeable external structural damage, including significant spalling of cement mortar and even aggregates. The limited amount of internally absorbed aqueous solution is insufficient to compensate for the effects of external damage, resulting in quality degradation at the onset of the F-T cycles. As the number of F-T cycles increases, the development of cracks hinders the continued absorption of external aqueous solution. The ongoing accumulation of F-T damage leads to the further detachment of cement mortar and aggregates from the specimens, resulting in a progressive decline in specimen quality [37,38].

#### 3.2.3. Relative Dynamic Modulus of Elasticity

**Figure 9:**
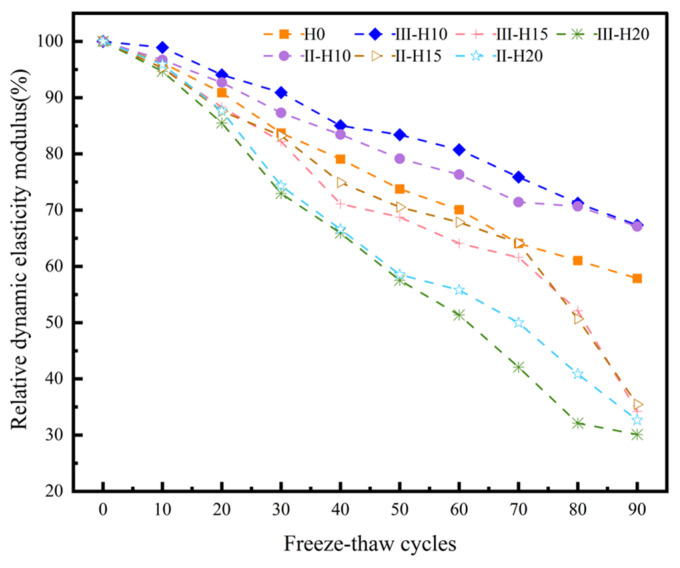
RDEM of concrete after F-T cycles.

Figure 9 illustrates the evolution of the relative dynamic elastic modulus(RDEM) of specimens subjected to progressive F-T cycles. Concrete matrices possess characteristic natural frequencies that undergo systematic modulation during cyclic F-T exposure due to cryogenic damage mechanisms. These mechanisms involve the nucleation and propagation of microcracks, as well as the coarsening of pore structures, which collectively degrade the material’s compactness [39]. As shown in Figure 9, all experimental groups exhibit a gradual decline in dynamic elastic modulus, which can be directly attributed to the cumulative deterioration effects caused by repetitive ice crystallization pressures and interfacial stress concentrations within the cementitious composites. Relative to the benchmark group, the degradation rate of the RDEMs for the two groups of specimens is lower when the HMSWIFA content is 10%. After 90 F-T cycles, the modulus degrades to 67.1% and 61.3% of the initial value, compared to degradation to 57.8% of the initial value in the benchmark group, indicating an improvement in the freezing resistance of the concrete specimens. However, as the HMSWIFA content increases, the RDEM of the concrete specimens compared to the reference group significantly decreases. Notably, at an HMSWIFA content of 20% after 90 F-T cycles, the RDEM of the specimens drops to only 30.1% and 32.6% of the initial value. This phenomenon occurs because a lower HMSWIFA content effectively fills small cracks and pores within the specimens, enhancing the compactness of the concrete. Conversely, when the HMSWIFA content exceeds a certain threshold, it can have detrimental effects, leading to increased porosity within the concrete. This results in a loosening of the internal structure, and as the HMSWIFA content continues to rise, the negative effects become progressively more pronounced.

### 3.3. Micro-Mechanism Analysis

**Figure 10:**
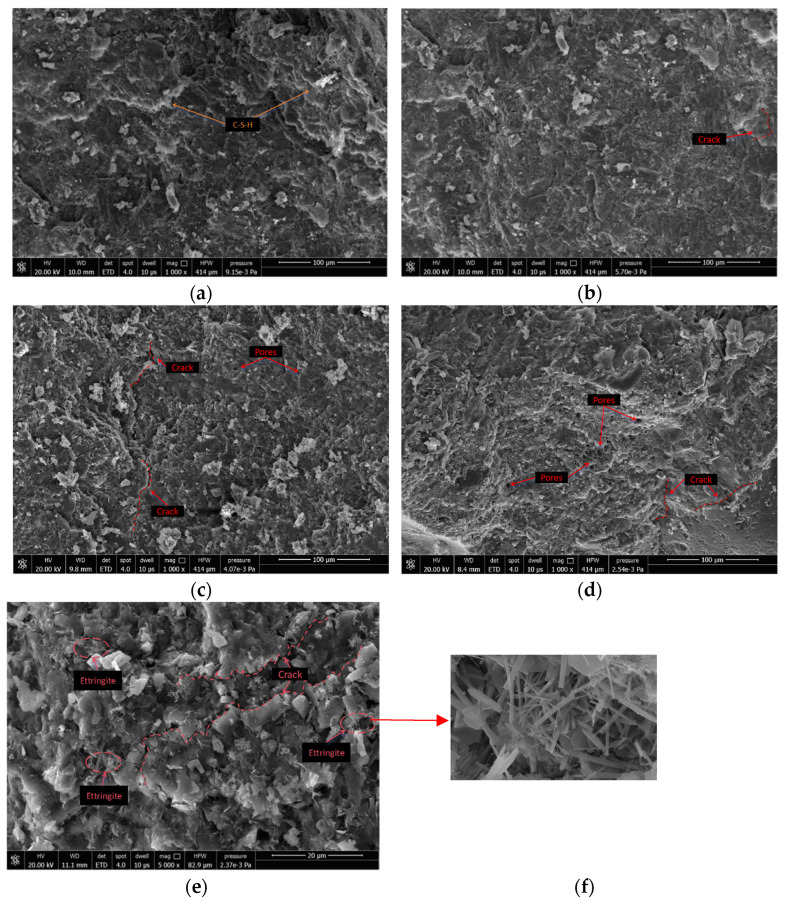
SEM images of different HMSWIFA substitution rates. (**a**) H0; (**b**) I-H10; (**c**) I-H15; (**d**) I-H20; (**e**) SEM images of ettringite and pore structure in I-H20; (**f**) SEM local enlargement.

To investigate the impact of HMSWIFA on the internal microstructure and hydration products of RCA, this study analyzed the microstructure of RCA specimens containing HMSWIFA at admixture levels of 0%, 10%, 15%, and 20% using Scanning Electron Microscopy (SEM). Figure 10 presents the SEM images of RCA with varying HMSWIFA content (0%, 10%, 15%, and 20%), and it can be seen from Figure 10a that, in the case of unadulterated HMSWIFA, the specimen is a relatively dense C-S-H gel structure, with no obvious cracks and pores, and the integrity of the integrity is intact; when the content of HMSWIFA is 10% (Figure 10b), the internal structure of the specimen is changed slightly, the C-S-H gel is in the form of a large piece, there are small cracks but no obvious pores, the integrity is still good, and it will have a negligible influence on the mechanical properties of the specimens, which is also one of the reasons why the CS of the concrete is still high under the condition of a small amount of HMSWIFA admixture (Figure 4); When the HMSWIFA content is 15% (Figure 10c), significant internal changes occur within the specimen. Holes of various sizes become apparent, and the structure gradually becomes more porous. The C-S-H gel is no longer continuous in small pieces, which undoubtedly reduces the integrity of the specimen and the load-bearing capacity, weakening the mechanical properties of the specimen; when the amount of HMSWIFA is 20% (Figure 10d), the internal structure is even more loose and porous and there are many large holes, and there are scattered pieces of small pieces of C-S-H gel, which implies that the integrality continues to decrease, the load-bearing capacity of concrete specimens is reduced and continues to decrease, and the bearing capacity and mechanical properties of the concrete specimens are further weakened.

With the increase in HMSWIFA admixture, a significant number of needle- and rod-shaped crystals appeared within the specimen. These crystals can be identified as ettringite by analyzing the hydration reactions of the material and the morphological characteristics of the crystals. Analysis of the composition of HMSWIFA reveals that it contains a certain amount of sulfate. The incremental incorporation of HMSWIFA leads to increasing sulfate ion concentrations within the cementitious matrix. These sulfate species chemically interact with calcium aluminate hydrates, initiating the crystallization of ettringite (AFt). Controlled ettringite formation provides beneficial pore-filling effects, enhancing concrete compactness and mechanical resistance through the optimization of pore structure [40]. However, excessive ettringite generation paradoxically increases matrix porosity through two synergistic mechanisms: (1) expansive crystallization pressures that induce microcrack nucleation, and (2) the enhancement of geometric complexity within void networks, despite initial pore occupation [41]. As illustrated in Figure 10e, when the internal expansive stresses induced by ettringite exceed the tensile strength threshold of concrete, interconnected fracture networks preferentially propagate along interfacial transition zones (ITZs). The progressive coalescence of cracks under sustained ettringite growth ultimately compromises both structural integrity and durability performance.

## 4. Conclusions

In this study, various RCA incorporating HMSWIFA were prepared. We conducted analyses of CS, FS, mass loss ratio, RDEM, and scanning electron microscopy (SEM) to investigate the effects of different particle sizes and content of HMSWIFA on the mechanical properties, frost resistance, and microstructure of RCA. The conclusions drawn from the study are as follows:

(1) HMSWIFA particle size and content significantly influence the mechanical properties of concrete. When the particle size of HMSWIFA ranges from 600 to 900 μm and the content is between 10% and 15%, the 28-day CS of RCA increases by 1.90% to 3.60%. However, the effect on FS is minimal, with a decrease of no more than 1.81%. As the particle size decreases or the mixing amount increases, the aluminum enrichment in HMSWIFA leads to increased porosity, which limits the formation of C-(A)-S-H gel and results in a deterioration of mechanical properties.
(2) A moderate amount of HMSWIFA (10% incorporation) can optimize frost resistance: the mass loss rate is reduced by 0.37% to 0.45% after 90 F-T cycles, and the RDEM is improved by 16.09% to 16.44%. This improvement is attributed to pore refinement and reinforcement of the interfacial transition zone. However, excessive incorporation (≥15%) significantly accelerates F-T damage due to the accumulation of calcite swelling stress and increased pore connectivity.
(3) A small amount of HMSWIFA admixture can effectively refine the pore structure of concrete specimens, enhancing their compactness and integrity, and thereby improving their overall performance. Microstructural analysis revealed that HMSWIFA effectively filled the pores and promoted densification at a content of 10%. However, at contents of 15% or greater, sulfate-induced excessive calcite generation, this phenomenon induces the propagation of microcracks and an escalation in porosity, ultimately leading to a decline in macroscopic properties.

## Figures and Tables

**Table 1:** Physical properties of the cement.

Initial Setting Time (min)	Final Setting Time (min)	CS (MPa) 3 d/28 d	FS (MPa) 3 d/28 d
183	248	22.3/47.4	4.9/7.8

**Table 2:** Chemical composition of the cement.

Chemical Composition	SiO_2_	CaO	Al_2_O	Fe_2_O_3_	MgO	Na_2_O	K_2_O
Content	21.65	61.88	5.35	3.21	2.52	0.21	0.16

**Table 3:** Physical properties of the ordinary coarse aggregate.

Aggregate Size (min)	Crushing Index (%)	Density (kg/m^3^)
5–26.5	15.2	2586.3

**Table 4:** Physical properties of the sand.

Mud Content (min)	Crushing Index (%)	Density (kg/m^3^)	Water Absorption(%)	Porosity (%)	Particle Size Modulus (mm)
0.9	8.2	2641.2	0.7	44.3	2.9

**Table 5:** Mix proportions of concrete (kg·m^−3^).

Specimen Number	Water	Cement	Sand	Natural Stone	RCA	HMSWIFA
H0	262.5	691.5	768	1502.4	375.6	0.00
I/II/III-H5	262.5	656.93	768	1502.4	375.6	34.575
I/II/III-H10	262.5	622.35	768	1502.4	375.6	69.15
I/II/III-H15	262.5	587.78	768	1502.4	375.6	103.73
I/II/III-H20	262.5	553.20	768	1502.4	375.6	138.30

**Table 6:** Test methods and related instruments.

Test Category	Test Age (d)	Specimen Size (mm)	Number of Specimens	Testing Instruments	Test Category
Cube compressive strength test	3/7/28	100 × 100 × 100	3	YAM-3000A Microcomputer Control Electro-hydraulic Servo Press Machine (NANJING TIMES TESTING EQUIPMENT Co., Ltd., Nanjing, China)	Cube compressive strength test
Flexural strength test	3/7/28	100 × 100 × 400	3	PWS-100 Electro-hydraulic servo static universal testing machine (Jinan Limei Electromechanical Technology Co., Ltd., Jinan, China)	Flexural strength test
Frost resistance test	28	100 × 100 × 400	3	TDR-9 Concrete Fast Freezing and Thawing Tester (Xinyuan Electromechanical Instrument Factory, Liaoning, China)	Frost resistance test
Dynamic modulus of elasticity test	28	100 × 100 × 400	6	DT-10W Dynamic Elastic Modulus Tester (Xin Gao Instrument Co., Ltd., Guizhou, China)	Dynamic modulus of elasticity test
Scanning electron microscope test	28	/	3	Field Emission Environmental Scanning Electron Microscope	Scanning electron microscope test

## Data Availability

The raw data supporting the conclusions of this article will be made available by the authors on request.
